# Endogenous and Exogenous Melatonin Exposure Attenuates Hepatic MT_1_ Melatonin Receptor Protein Expression in Rat

**DOI:** 10.3390/antiox8090408

**Published:** 2019-09-18

**Authors:** Alexander M. Mathes, Paul Heymann, Christian Ruf, Ragnar Huhn, Jochen Hinkelbein, Thomas Volk, Tobias Fink

**Affiliations:** 1 Department of Anesthesiology and Intensive Care Medicine, University Hospital Cologne, Kerpener Str. 62, 50937 Cologne, Germany; jochen.hinkelbein@uk-koeln.de; 2 Department of Anesthesiology, Critical Care and Pain Medicine, Saarland University Hospital, 66424 Homburg, (Saar), Germany; heymann.paul@gmail.com (P.H.); thomas.volk@uks.eu (T.V.); tobias.fink@uks.eu (T.F.); 3 Department of Urology, Bundeswehrkrankenhaus Ulm, Oberer Eselsberg 40, 89081 Ulm, Germany; christruf@gmx.de; 4 Department of Anesthesiology, University Hospital Duesseldorf, Moorenstr. 5, 40225 Duesseldorf, Germany; Ragnar.Huhn@med.uni-duesseldorf.de

**Keywords:** melatonin receptor, liver, spatial distribution, melatonin, ramelteon, shock

## Abstract

Melatonin receptors are highly relevant for the hepatoprotective effects of the pineal hormone melatonin after experimental hemorrhagic shock in rats. In this study, we sought to determine the spatial expression pattern and a putative regulation of two melatonin receptors, membrane bound type 1 and 2 (MT_1_ and MT_2_), in the liver of rats. In a male rat model (Sprague Dawley) of hemorrhage and resuscitation, we investigated the gene expression and protein of MT_1_ and MT_2_ in rat liver by utilizing real-time quantitative polymerase chain reaction, a western blot analysis, and immunohistochemistry. Plasma melatonin content was measured by an enzyme-linked immunosorbent assay. Male rats underwent hemorrhage and were resuscitated with shed blood and a Ringer’s solution (*n* = 8 per group). After 90 min of hemorrhage, animals were given vehicle, melatonin, or ramelteon (each 1.0 mg/kg intravenously). Sham-operated controls did not undergo hemorrhage but were treated likewise. Plasma melatonin was significantly increased in all groups treated with melatonin and also after hemorrhagic shock. Only MT_1_, but not the MT_2_ messenger ribonucleic acid (mRNA) and protein, was detected in the rat liver. The MT_1_ protein was located in pericentral fields of liver lobules in sham-operated animals. After hemorrhagic shock and treatment with melatonin or ramelteon, the hepatic MT_1_ protein amount was significantly attenuated in all groups compared to sham controls (50% reduction; *p* < 0.001). With respect to MT_1_ mRNA, no significant changes were observed between groups (*p* = 0.264). Our results indicate that both endogenous melatonin exposure from hemorrhagic shock, as well as exogenous melatonin and ramelteon exposure, may attenuate melatonin receptors in rat hepatocytes, possibly by means of desensitization.

## 1. Introduction

The hepatoprotective potential of melatonin administration has been well investigated in the past. In various models of stress, therapy with melatonin has been demonstrated to reduce both hepatocellular damage and liver dysfunction. Hepatic injury following hemorrhagic shock [1,2], pancreatitis [3], ischemia and reperfusion [4], and a number of toxic challenges, such as methanol [5], cadmium [6] or carbon tetrachloride [7,8], may successfully be treated with different regimes of melatonin preconditioning and treatment.

Melatonin’s beneficial effects are usually attributed to the specific and high direct antioxidant capacity of the pineal hormone [9], although melatonin may also exhibit pro-oxidative or even cytotoxic effects under certain circumstances [10]. However, recent evidence has suggested that the protective potential of the pineal hormone may also rely on melatonin receptor activation. In the heart [11,12], the gut [13], the liver [1,2] and the brain [14], the protective effect of melatonin has been abolished by a novel melatonin receptor antagonist (luzindole), indicating the importance of the melatonin receptor pathway. Furthermore, we demonstrated that the melatonin receptor agonist ramelteon, a drug against insomnia and also a melatonin receptor agonist, may effectively attenuate the influence of hemorrhagic shock on liver function and hepatic perfusion in rats, despite having no direct antioxidant effects on its own [15].

Melatonin and its receptor messenger ribonucleic acid (mRNA) have been identified in the livers of rats [16,17], mice [18], and various other mammals. However, there are conflicting data regarding the question of which subtypes of membrane bound melatonin receptors type 1 and 2 (MT_1_ and MT_2_) may be present in rat hepatocytes [16,17]. Several studies have demonstrated that human melatonin receptors may not only be desensitized but also even internalized after exposure to physiological or supraphysiological levels of melatonin in vitro [19,20,21]. Furthermore, melatonin exposure may influence genetic expression patterns in rat livers [22]. However, the spatial allocation of melatonin receptors in liver lobules is unknown, and regulatory changes in the expression of hepatic melatonin receptors after melatonin receptor agonist exposure have, to our knowledge, not been investigated in an in vivo model. Therefore, this study was designed to determine the hepatocellular and regional expression pattern, as well as mRNA and protein expression, of hepatic melatonin receptor subtypes MT_1_ and MT_2_ in male rats exposed to either physiologically generated melatonin following hemorrhage (endogenous) or to therapy with melatonin or ramelteon (exogenous) in sham-operated animals and after hemorrhagic shock.

## 2. Material and Methods

### 2.1. Drugs and Chemicals

Ramelteon and melatonin were dissolved in dimethyl sulfoxide (DMSO) to final concentrations of 0.1% for animal experiments. DMSO was not higher than 5.0% in the final solution. All chemicals were purchased from a national distributor of Sigma (Sigma-Aldrich, Munich, Germany), unless specified different in the following.

### 2.2. Animals

Animal experiments were approved by the responsible regional committee on animal use (LGV, Saarbrücken, Germany; permission no. 31/2006, 16/2007) and were carried out as specified by the German Animal Welfare Act. Rats (male, 200–300 g body weight, Sprague Dawley) were obtained from the national distributor of Charles River (Sulzfeld, Germany). There was free access to food and water for all animals; however, twelve hours prior to surgery, pellet food was withheld. In the time before the experiments, rats were entrained to a circadian rhythm (equal light–dark cycle of 12:12 h). To avoid influences of endogenous melatonin, all experiments began at the same time (Zeitgeber time 2 h).

### 2.3. Surgical Procedures

All surgical and interventional procedures were performed as published previously [1]. Rats were anesthetized by an injection of a hypnotic (sodium pentobarbital 50 mg/kg) in the left lower abdomen (intraperitoneally). To allow for spontaneous breathing, an open tracheotomy was performed, and a polyethylene (PE) tube inserted. For infusions, the right external jugular vein was catheterized with a PE catheter, and for the measurement of mean arterial pressure (MAP) and heart rate (HR) the A. carotis sinistra was catheterized (Monitor Modul 66S, Hewlett Packard, Palo Alto, CA, USA). For hemorrhagic shock, a pressure controlled model was used; rapid arterial blood withdrawal over 5 min via the carotid artery was performed to reach an MAP of 35 ± 5 mm Hg for 90 min. For resuscitation, rats were given 60% of their individual shed blood volume (injection via the central vein over 5 min), followed by a reperfusion time of two hours. For reperfusion, the fluid management consisted of 200% of each rat’s individual shed blood volume as a Ringer’s solution in the first hour, followed by 100% in the second hour of reperfusion. At baseline, at the end of hemorrhagic shock, as well as at the end of the experiment (after 2 h of reperfusion), a blood gas analysis (BGA) was performed. For the BGA, we used 0.2 mL blood withdrawn from the A. carotis sinistra in a commercially available BGA machine (pHOx plus L, nova biomedical, Mörfelden-Walldorf, Germany). After reperfusion, animals underwent a laparotomy for isolated liver perfusion via the portal vein with 20 mL of normal saline. The liver was harvested, dissected into three parts, and immediately transferred to RNAlater (Qiagen, Hilden, Germany), liquid nitrogen, or formalin for later processing. Animals were sacrificed by the rapid injection of lethal of pentobarbital after the removal of the liver.

### 2.4. Experimental Protocol

**Figure 1:**
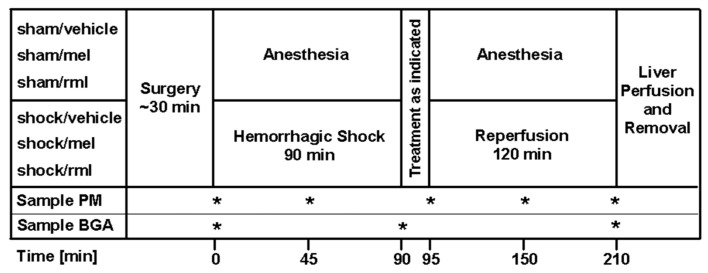
After the induction of anesthesia, groups were treated as indicated (*n* = 8 per group). Samples for blood gas analyses (BGA) and plasma melatonin content (PM) were taken at the times marked with an asterisk. rml = ramelteon; mel = melatonin.

For an overview of the experimental protocol, please refer to Figure 1. Sham-operated controls were intravenously given 10 mL/kg/h of a Ringer’s solution and vehicle DMSO, but no hemorrhagic shock was induced (sham/vehicle; *n* = 8). Two groups of sham-operated animals also intravenously received ramelteon or melatonin of 1.0 mg/kg instead of vehicle DMSO (sham/rml and sham/mel, *n* = 8 each). One group underwent hemorrhagic shock and resuscitation and was injected vehicle DMSO at the time of reperfusion (shock/vehicle; *n* = 8). For ramelteon and melatonin therapy, animals were intravenously treated with 1.0 mg/kg of either ramelteon or melatonin immediately after hemorrhagic shock at the time of reperfusion (shock/rml and shock/mel; *n* = 8 each).

### 2.5. Enzyme-Linked Immunosorbent Assay

In the course of the experiment, plasma melatonin content was measured at baseline (after surgical procedures), as well as after 45/95/150 and 210 min, by means of an enzyme-linked immunosorbent assay (ELISA) using a commercial grade kit (Melatonin ELISA, IBL International GmbH, Hamburg, Germany).

### 2.6. RNA Isolation

Hepatic tissues with approximately 70–80 mg from every animal (*n* = 8 per group) were stored in a RNAlater solution (Qiagen, Hilden, Germany) at −20 °C, thawed, homogenized (Homogenizer, Omni International, Kennesaw, GA, USA), and digested using proteinase K (concentration: 20 mg/mL; purchased from Invitrogen, Karlsruhe, Germany). Total RNA was isolated using the RNeasy Mini kit (Qiagen, Hilden, Germany), and remaining DNA was digested using the RNase free DNase Set (Qiagen, Hilden, Germany), as specified in the instructions of manufacturer. From each sample of tissue, approximately 140 µg of total RNA was isolated. RNA was stored at −80 °C until real-time quantitative polymerase chain reaction (RTQ-PCR) was performed. To assure the quality and purity of isolated total RNA, we performed spectral photometry, agarose gel electrophoresis, further PCR (using β-Actin primers for detecting DNA contamination), and finally microfluidics-based quality checks with an Agilent Bioanalyzer 2100 (Agilent, Waldbronn, Germany). We employed only total RNA without a detectable contamination of DNA in the PCR analysis, with a RNA integrity number >7, as well as with a ratio A260/A280 >1.9 as analyzed using spectral photometry, with 28S ribosomal bands that were detectable at approximately twice the amounts of the 18S RNA in agarose gel electrophoresis.

### 2.7. RTQ-PCR

Each 5 µg aliquot of total RNA was reverse transcribed over two hours using a two-step PCR protocol and by applying the “High Capacity cDNA Reverse Transcription Kit” according to the manufacturer’s specifications (Applied Biosystems, Weiterstadt, Germany). Each 10 µL of diluted complementary deoxyribonucleic acid (cDNA) contained 10 ng of an RNA equivalent in the first evaluation and 0.5 µg of an RNA equivalent in the second evaluation; this cDNA was used as a template for the following PCR reaction. The relevant sequences for gene targets were identified, and a rat-specific inventoried primer and probe design for MT_1_ (Rn01488022_m1) and MT_2_ (Rn01447987_m1) were utilized for RTQ-PCR. TaqMan^®^ Universal PCR Master Mix and the GeneAmp 5700 Sequence Detection System (SDS, Version 1.3, TaqMan) were used. For the detection of eukaryotic 18S rRNA (4310893E), an assay was used for normalization; furthermore, a relative standard curve allowed for quantification purposes. Typically, the dynamic range of linearity lasted over 6 log scales. All materials for RTQ-PCR were purchased from Applied Biosystems (Weiterstadt, Germany).

### 2.8. Western Immunoblot Analysis

For the western immunoblot analysis, 100 mg of hepatic tissue were homogenized in 10 volumes of a cell lysis buffer. Homogenates were clarified by centrifugation at 14,000× *g*, and total soluble protein concentration was measured using a modified Bradford test. Aliquots of protein, using 100 µg per lane, were fractionated by gel electrophoresis (sodium dodecyl sulfate-polyacrylamide) under denaturing conditions, and a tris(hydroxymethyl)-aminomethan (TRIS)-glycine- buffer system in 12% TRIS-glycine gels was applied (Anamed, Offenbach, Germany). The gels were then electroblotted to polyvinylidenfluorid western blotting membranes (Roche, Mannheim, Germany) and kept at 4 °C; antigen detection was performed within 24 h. By preincubation with 5% Slimfast^®^ (Kainos, Dallas, TX, USA) in TRIS-buffered saline/Tween (20 mmol/L TRIS [pH 7.5], 0.5 mol/L NaCl, 0.1% Tween 20), nonspecific binding sites were blocked. The incubation of the membrane was performed with polyclonal goat antirat MT_1_ or antimouse MT_2_ antibodies (dilution 1:1000; MT_1_: sc-13186; MT_2_: sc-13177; Santa Cruz Biotechnology Inc., Santa Cruz, CA, USA). By washing the membrane with TRIS-buffered saline/Tween, the unbound primary antibody was removed. A horse radish peroxidase-linked donkey anti-goat antibody (dilution 1:10,000; sc-2020; Santa-Cruz Biotechnology Inc., Santa Cruz, CA, USA) was used as the secondary antibody. By using an enhanced chemiluminescent reaction using the ECL Western Blot Analysis System (Amersham Buchler, Braunschweig, Germany), the detection of the antigen–antibody conjugate was realized. By short exposure to a blue-light sensitive autoradiography film (Fuji Medical X-Ray Film, Fujifilm Europe, Düsseldorf, Germany), the signal was detected and analyzed by densitometry.

### 2.9. Immunohistochemical Staining

Formalin-fixed, paraffin-embedded, and further dewaxed liver sections were used to evaluate the regional and cell-specific expressional pattern of MT_1_ and MT_2_ after 2 h of reperfusion. Using microwave irradiation, sections were exposed to antigen retrieval. By incubation in 3% H_2_O_2_-methanol, endogenous peroxidase activity was blocked. After subsequent treatment with normal rabbit serum, slides were incubated at 37 °C for 1 h with polyclonal goat antirat MT_1_ or antimouse MT_2_ primary antibodies (dilution 1:200; MT_1_: sc-13186; MT_2_: sc-13177; Santa Cruz Biotechnology Inc., Santa Cruz, CA, USA Santa Cruz Biotechnology, Inc., Santa Cruz, CA). A secondary antibody (biotinylated rabbit anti-goat antibody) was used for staining with streptavidine–biotin complex peroxidase. For chromogens, 3,3′-diaminobenzidine and 3% CoCl_2_ were used, and slides were counterstained with hematoxylin.

### 2.10. Statistical Analysis

Parametric data are expressed as means ± standard deviation (SD), and non-parametric data are expressed as median ±25th/75th percentile. Statistical evaluation was performed with SigmaPlot^®^ 9.0 with SigmaStat integration (Systat Software, Erkrath, Germany). After the evaluation of normal distribution, a one-way analysis of variance (ANOVA) followed by a Student–Newman–Keuls test was used for parametric data, while Kruskal–Wallis ANOVA followed by a Dunn’s test was used for non-parametric data; repeated measures the ANOVA were applied for hemodynamic data and plasma melatonin content, when applicable; *p* < 0.05 was considered statistically significant.

## 3. Results

### 3.1. Hemodynamic Parameters and Analysis of Blood Gases

**Figure 2:**
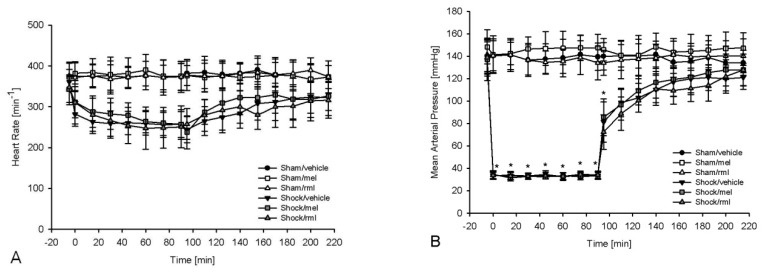
The recovery of the heart rate (**A**) and mean arterial pressure (**B**) was equivalent in all shock groups after resuscitation. No significant difference was detected between groups that underwent a hemorrhage. An asterisk (*) indicates *p* < 0.05 vs. baseline values. Data are expressed as mean ± SD (*n* = 8). rml = ramelteon; mel = melatonin.

In all groups, the corresponding baseline values for MAP and heart rate (Figure 2A,B) and for blood gas analysis (Table 1) were comparable. The sham, melatonin and ramelteon control groups had stable hemodynamics throughout the whole experiment. A hemorrhage resulted in a typical and non-significant decrease of heart rate. There was a significant reduction of hemoglobin content and base excess, as well as a significant increase in lactate levels after hemorrhagic shock (*p* < 0.05 vs. baseline). In all shock groups, hemorrhage was reversible, and hemoglobin content, lactate levels and base excess recovered significantly (*p* < 0.05 vs. end of shock). Body weight-adapted shed blood volumes were not different between shock groups (shock/vehicle 48.33 ± 4.09 mL/kg; shock/mel 46.93 ± 4.56 mL/kg; shock/rml 46.71 ± 5.22 mL/kg; *p* = 0.754), indicating a comparable insult.

### 3.2. Plasma Melatonin Content

During the course of the experiment, plasma melatonin content was in the physiological daytime range in sham-operated animals treated with vehicle or ramelteon (Table 2). After hemorrhagic shock, plasma melatonin amount was significantly increased at 95 min, i.e., 5 min after reperfusion, in vehicle- and ramelteon-treated animals (*p* < 0.001 for shock/vehicle and shock/rml vs. sham/vehicle, sham/rml, sham/mel and shock/mel); no significant increase was noted at time points 150 or 210 min. In sham and shock animals receiving melatonin, plasma melatonin content was significantly increased to high levels at 95/150/210 min compared to all other groups (*p* < 0.001 for sham/mel and shock/mel vs. sham/vehicle, sham/rml, shock/vehicle and shock/rml). The power of the performed tests was 1.0 (α = 0.05).

### 3.3. Melatonin Receptor mRNA

**Figure 3:**
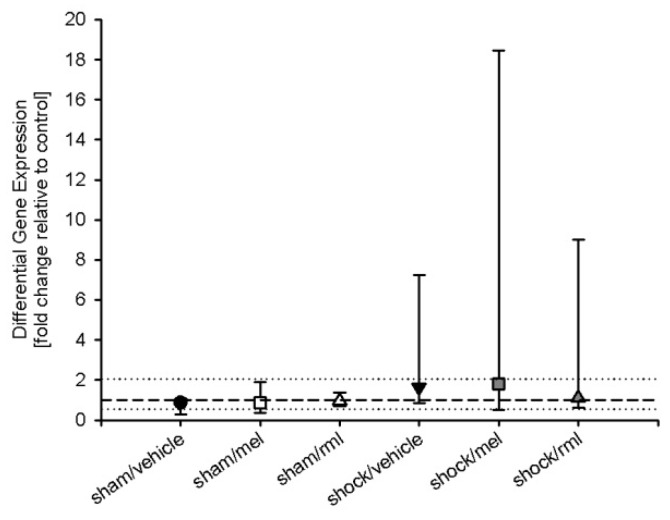
Analysis of differential expression of melatonin receptor type 1 messenger ribonucleic acid displayed no significant differences between groups. The dashed line indicates the mean of sham/vehicle; the dotted lines indicate a ratio of 0.5 for downregulated genes and 2.0 for upregulated genes. Data are expressed as median ±25th/75th percentile (*n* = 8). rml = ramelteon; mel = melatonin.

RTQ-PCR indicated the presence of MT_1_ mRNA but not of MT_2_ mRNA in rat liver. MT_2_ mRNA measurements were outside the linear dynamic range of RTQ-PCR and could be not detected (data not shown). A high concentration of RNA equivalents (0.5 µg) had to be used for RTQ-PCR to detect MT_1_ mRNA. For MT_1_, differential gene expression, defined as the ratio of normalized gene expression relative to control (sham/vehicle) with a ratio >2 (for upregulated genes) or <0.5 (for down-regulated genes), did not differ between groups (*p* = 0.264) (Figure 3). The power of the performed tests was 0.225 (α = 0.05).

### 3.4. Melatonin Receptor Protein

**Figure 4:**
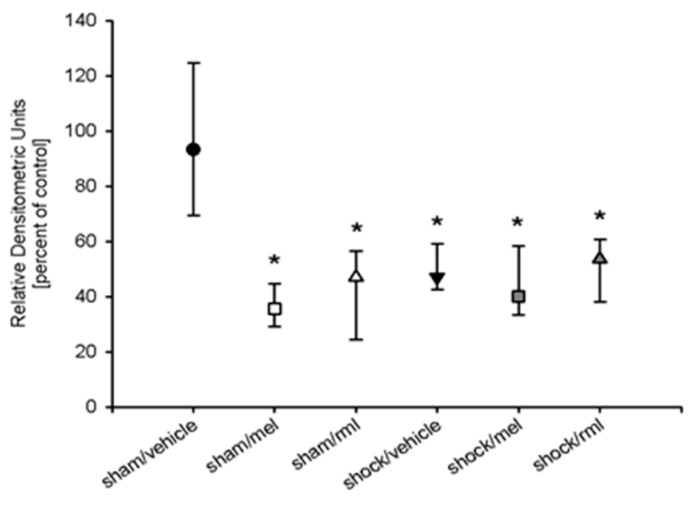
Densitometric analysis of western blotting of melatonin receptor type 1 protein. The analysis revealed a significant attenuation in all groups that underwent hemorrhagic shock and/or treatment with melatonin or ramelteon, compared to vehicle controls. An asterisk (*) indicates *p* < 0.01 vs. sham/vehicle. Data are expressed as median ±25th/75th percentile (*n* = 8). rml = ramelteon; mel = melatonin.

The western immunoblot analysis revealed the presence of the MT_1_ protein but not of the MT_2_ proteins in rat livers. Compared to sham-operated animals, the amount of MT_1_ protein was significantly reduced in all animals treated with either melatonin or ramelteon, as well as those undergoing hemorrhagic shock (approximately 50% reduction; *p* < 0.001 vs. sham/vehicle) (Figure 4). No difference was noted between groups that were exposed to melatonin or ramelteon. The power of the performed tests was 0.99 (α = 0.05).

### 3.5. Spatial Expression Pattern of Melatonin Receptors

**Figure 5:**
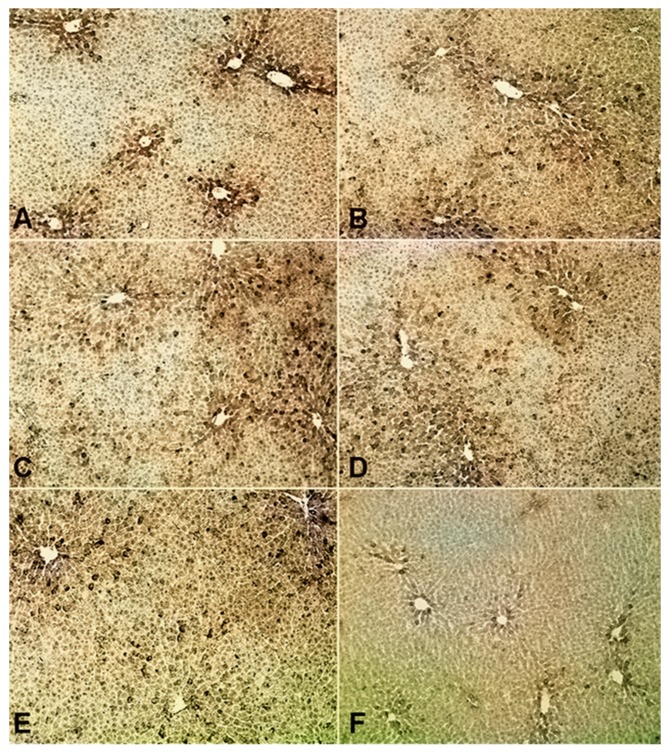
Representative images of liver sections obtained from sham-operated animals treated with vehicle (**A**), melatonin (**B**) or ramelteon (**C**), as well from animals that underwent hemorrhagic shock and treatment with vehicle (**D**), melatonin (**E**) or ramelteon (**F**). Melatonin receptors type 1 were located in a dense composition in pericentral areas of liver lobules in vehicle treated animals; this pattern was replaced by a diminished receptor expression around the central vein after hemorrhagic shock and/or treatment with melatonin or ramelteon. Hematoxylin-stained; magnification 100×.

An immunohistochemical analysis indicated the presence of the MT_1_ protein but not of the MT_2_ protein, in the rat liver. In sham-operated animals, MT_1_ was found in a dense configuration around the central veins of hepatic lobules, while hardly any MT_1_ was identified in the periportal regions of hepatic lobules (Figure 5A). This configuration was altered after treatment with melatonin (Figure 5B) or ramelteon (Figure 5C), as well as in all groups undergoing hemorrhagic shock (Figure 5D–F), resulting in a somewhat disseminated pattern of MT_1_ in the centrilobular area in all groups. After hemorrhagic shock and treatment with melatonin (Figure 5E) or ramelteon (Figure 5F), the presence of MT_1_ was diminished.

## 4. Discussion

This study identified the spatial expression pattern of hepatocellular melatonin receptors in rats and indicated an attenuation of hepatic melatonin receptors after endogenous melatonin exposure following hemorrhagic shock, as well as after exogenous melatonin or ramelteon administration. Plasma melatonin levels were significantly increased after hemorrhage and reperfusion, as well as after exogenous melatonin treatment. Melatonin receptor mRNA and protein were detected for MT_1_ but not for MT_2_ in the rat liver. A dense configuration of MT_1_ was found primarily in the central lobular region of the liver. The spatial distribution of MT_1_ was altered by the induction of hemorrhagic shock and resuscitation, as well as after treatment with melatonin or ramelteon, resulting in a disseminated composition in pericentral areas. Furthermore, the amount of the hepatic MT_1_ protein was significantly reduced after hemorrhage and therapy with melatonin or ramelteon. With respect to MT_1_ mRNA, we detected no significant changes between groups.

The endogenous release of melatonin after hemorrhagic shock, as demonstrated in this study, is similar to the results obtained in mice by Wichmann and colleagues [23] and represents an increase of melatonin plasma concentrations about 20 times the amounts typically found during the night. However, our data show that the endogenous rise in melatonin plasma levels after hemorrhage and resuscitation remained significant for only a short period of time; after 55 min, the elevation was no longer detectable. After exogenous treatment, melatonin plasma levels remained significantly increased for the whole time of reperfusion and were significantly higher compared to endogenous release. This indicates that endogenous release was significant, but the endogenous release lasted much longer, as plasma levels were significantly higher.

Melatonin receptors, as identified by immunohistochemistry, appeared to be distributed in a heterogeneous pattern in the liver of sham-operated rats. While MT_1_ was found in a dense composition in hepatocytes around the central veins, hardly any MT_1_ was identified in the periportal area of liver lobules. This finding is partially in line with previous reports on a differential expression pattern of melatonin receptors in the chicken liver [24]. As a consequence of their specific metabolic state, pericentral fields of the liver are highly sensitive to ischemic stress as compared to the periportal areas, which are slightly better oxygenated [25]. Thus, hemorrhagic shock quickly leads to a characteristic pattern of central lobular necrosis [26]. A differential allocation of hepatic melatonin receptors could therefore allow for a specific focus of melatonin receptor-associated liver protection on areas in need. It is tempting to speculate that this pattern of MT_1_ expression might allow for a selective protection of centrilobular hepatocytes.

The spatial arrangement of MT_1_, as observed in sham-operated animals, was partially disrupted by the administration of melatonin or ramelteon and by the induction of hemorrhagic shock. The concentrated distribution of MT_1_ in hepatic pericentral areas, as found in untreated animals, was replaced by a scattered pattern in the pericentral field of liver lobules in all groups exposed to melatonin or ramelteon. This finding suggests that the observed changes in MT_1_ distribution are unlikely to be caused by metabolic disturbances like hypoxia or ischemia; the parameters of centrilobular distress, like the enhanced expression of glutathione synthetase-1, usually show an expansion from the first pericentral cellular layer into midzonal areas after hemorrhagic shock and resuscitation [27]. Thus, centrilobular cell damage would have attenuated MT_1_ around the central vein, and not in a disseminated fashion, as demonstrated in this study.

In all animals that were exposed to melatonin or ramelteon, we observed a significantly reduced the amount of the MT_1_ protein in the liver compared to the sham-operated animals. With respect to MT_1_ mRNA, no statistically significant changes were noted between groups. This indicates that our findings are not attributable to an altered expression of melatonin receptor genes, but they may indicate post-transcriptional modifications or receptor desensitization. However, it needs to be acknowledged that the total amount of MT_1_ mRNA was extremely small in all samples investigated, and large inter-individual variations were observed within each treatment group, especially in all shock groups. This was also reflected by a low power of the underlying statistical analysis. Thus, it appears possible that MT_1_ mRNA variations may have been present but were not detected by our means.

Since we observed larger inter-individual variations of MT_1_ mRNA in all shock groups compared with sham-operated animals, the question whether other factors of hemorrhagic shock may have influenced our results must be considered. Hemorrhage and resuscitation may have a significant influence on hepatic inflammatory gene expression [28,29] and have been demonstrated to modify hepatocellular receptor expression [30]. However, the MT_1_ receptor protein was attenuated in all groups exposed to melatonin or ramelteon, even in groups that did not undergo hemorrhagic shock. This indicates that melatonin exposure is likely to be the relevant factor in attenuating hepatic MT_1_ expression, but hemorrhage and resuscitation are not.

With respect to the MT_2_ protein, it needs to be noted that to our knowledge, no rat specific MT_2_ antibody is available; as a consequence, an antimouse antibody with a high antirat cross-reactivity was used in this study. Therefore, we cannot exclude the possibility that the MT_2_ protein may be present in the liver of the Sprague Dawley rat. However, our failure to detect MT_2_ protein correlates with our results regarding MT_2_ mRNA, and control experiments with brain sections showed that our method is suitable to detect the MT_2_ protein in other tissues (data not shown in manuscript). Though the presence of hepatic MT_2_ mRNA has been described in the past [16,17,18], the corresponding protein has never been shown. Further, conflicting data are available regarding the presence of both subtypes of melatonin receptors in the liver [16,17,25]. Though these discrepancies cannot easily be explained, the available evidence suggests that if hepatic melatonin receptor MT_2_ mRNA and/or protein should be present, amounts are likely to be extremely small.

Our investigation was further limited to the extent that melatonin receptor antibodies have been controversially discussed in the past. Some researchers believe that certain antibodies may not always be highly specific. On the other hand, our results regarding the existence of hepatic MT_1_ receptors in rats are not only in line with previous investigations, but more importantly, they also show the same spatial pattern [24]. Furthermore, this antibody has been successfully used in a variety of previous investigations without dissent [31,32,33]. Therefore, despite all questions arising, we believe that our work contributes important and novel insight in hepatic melatonin receptors, even if the protein level needs to be interpreted with caution.

Our findings regarding the attenuation of melatonin receptors after melatonin receptor agonist exposure are partially in line with previous reports on the desensitization or internalization of melatonin receptors following melatonin administration in vitro [19,20,21]. Even a short treatment of 10 min of melatonin exposure was demonstrated to decrease iodomelatonin binding in recombinant human MT_1_ receptors and to desensitize recombinant human MT_2_ receptors in Chinese hamster ovary cells [20]. Human recombinant MT_1_ internalization was observed in the same cell line after a one hour treatment with melatonin [19]. For MT_1_ internalization, the C-terminal domain appears to be of high relevance, as a truncation of this tail may inhibit internalization processes [34]. With respect to our study, it is interesting to note that MT_1_ attenuation is not limited to the exposure of melatonin itself, as it may be induced by the administration of a melatonin receptor agonist, ramelteon.

Though hepatic melatonin receptors appear to play a significant role in organ protection after hemorrhagic shock [1,2,15] and seem to be involved in regulating hepatic glucose metabolism [35,36], their physiological significance remains to be discovered. Melatonin administration itself has proven to be highly beneficial after liver injury [1,2,3,4,5,6,7,8], like many other antioxidants [37,38,39,40,41]. For melatonin, a mechanism of receptor internalization could, in theory, induce parts of these beneficial changes in hepatocellular structures: MT_1_ internalization may have significant effects on cell morphology and, potentially, on gene expression [42]. In hepatocytes, receptor internalization appears to be a relevant mechanism in regulating hormone signaling [43,44]. It is therefore tempting to speculate that melatonin receptor internalization may also act as a regulatory tool for hepatic function.

## 5. Conclusions

We would like to conclude that hepatic MT_1_ are primarily localized in pericentral areas of liver lobules, suggesting a possible preferential protection of centrilobular cells. Both endogenous and exogenous melatonin exposure resulted in a significant attenuation of theMT_1_ protein, with a concomitant disruption of the spatial arrangement of MT_1_ in pericentral fields. As no significant changes of MT_1_ mRNA were detected in this study, we would like to suggest a process of the desensitization of hepatic MT_1_ following melatonin receptor agonist exposure in rats.

## Figures and Tables

**Table 1:** Blood gas analysis.

Group	Baseline Values	End of Shock	End of Experiment
		Hb [g/dL]	
sham/vehicle	10.7 ± 0.4	10.1 ± 0.4	10.1 ± 0.5
sham/mel	10.9 ± 0.4	10.8 ± 0.4	10.3 ± 0.6
sham/rml	11.2 ± 0.4	10.6 ± 0.4	10.1 ± 0.4
shock/vehicle	10.3 ± 0.3	* 6.4 ± 0.5	# 8.4 ± 0.4
shock/mel	11.2 ± 0.4	* 6.3 ± 0.4	# 8.7 ± 0.4
shock/rml	10.7 ± 0.3	* 6.6 ± 0.4	# 8.5 ± 0.5
		Lactate [mmol/L]	
sham/vehicle	1.7 ± 0.2	1.6 ± 0.3	1.7 ± 0.1
sham/mel	1.5 ± 0.3	1.8 ± 0.03	1.7 ± 0.4
sham/rml	1.6 ± 0.3	1.7 ± 0.3	1.5 ± 0.3
shock/vehicle	1.5 ± 0.3	* 8.5 ± 0.6	# 1.9 ± 0.3
shock/mel	1.9 ± 0.2	* 9.0 ± 0.6	# 2.0 ± 0.2
shock/rml	1.5 ± 0.3	* 7.7 ± 0.8	# 1.6 ± 0.4
		pH	
sham/vehicle	7.37 ± 0.02	7.39 ± 0.02	7.40 ± 0.02
sham/mel	7.37 ± 0.02	7.39 ± 0.02	7.42 ± 0.02
sham/rml	7.36 ± 0.02	7.39 ± 0.03	7.41 ± 0.03
shock/vehicle	7.37 ± 0.02	7.27 ± 0.02	7.34 ± 0.03
shock/mel	7.37 ± 0.02	7.28 ± 0.03	7.34 ± 0.03
shock/rml	7.37 ± 0.02	7.26 ± 0.03	7.35 ± 0.03
		BE [mmol/l]	
sham/vehicle	−1.6 ± 0.8	−1.8 ± 0.5	−3.8 ± 1.2
sham/mel	−1.2 ± 1.1	−1.9 ± 1.0	−4.1 ± 0.9
sham/rml	−1.0 ± 1.3	−1.9 ± 1.1	−3.3 ± 1.7
shock/vehicle	−1.6 ± 1.2	* −11.4 ± 2.0	# −6.1 ± 1.8
shock/mel	−.2 ± 1.1	* −11.9 ± 1.8	# −5.9 ± 1.2
shock/rml	−1.8 ± 0.9	* −12.3 ± 1.8	# −6.4 ± 1.5

Blood gas parameters. Analysis revealed normal baseline values for hemoglobin (Hb, g/dL), lactate (mmol/L), pH, and base excess (BE, mmol/L) in all groups. Hemorrhagic shock and recovery was significant and equivalent between groups. An asterisk (*) indicates *p* < 0.05 vs. baseline values, and a pound sign (^#^) indicates *p* < 0.05 vs. values at the end of shock. Data are expressed as mean ± SD (*n* = 8). rml = ramelteon; mel = melatonin.

**Table 2:** Plasma melatonin content [ng/mL].

Group	Baseline	45 min	95 min	150 min	210 min
sham/vehicle	0.07 ± 0.04	0.05 ± 0.05	0.15 ± 0.10	0.12 ± 0.05	0.05 ± 0.04
sham/mel	0.06 ± 0.04	0.04 ± 0.02	**17.90 ± 1.81 ^#^**	**11.39 ± 1.6 ^#^**	**1.19 ± 0.52 ^#^**
sham/rml	0.09 ± 0.10	0.08 ± 0.05	0.10 ± 0.04	0.11 ± 0.04	0.08 ± 0.06
shock/vehicle	0.04 ± 0.04	0.10 ± 0.05	1.62 ± 0.76 *	0.05 ± 0.05	0.04 ± 0.01
shock/mel	0.07 ± 0.06	0.05 ± 0.04	**18.47 ± 1.43 ^#^**	**11.80 ± 1.30 ^#^**	**2.25 ± 0.70 ^#^**
shock/rml	0.05 ± 0.04	0.04 ± 0.04	1.90 ± 0.54 *	0.04 ± 0.05	0.10 ± 0.01

Hemorrhagic shock resulted in significantly increased melatonin plasma amounts (in ng/mL) in vehicle (shock/vehicle) and ramelteon (shock/rml) treated groups, while melatonin administration led to supraphysiological levels of plasma melatonin in both groups (sham/mel and shock/mel). Significant changes are bold: An asterisk (*) indicates *p* < 0.001 for shock/vehicle and shock/rml vs. sham/vehicle, sham/rml, sham/mel and shock/mel. A pound sign (^#^) indicates *p* < 0.001 for sham/mel and shock/mel vs. sham/vehicle, shock/vehicle, sham/rml and shock/rml. Data are expressed as mean ± SD (*n* = 8). rml = ramelteon; mel = melatonin.
